# Physiologic assessment of CMR strain: afterload and contractility

**DOI:** 10.1186/1532-429X-18-S1-P48

**Published:** 2016-01-27

**Authors:** Tazim Merchant, Nathaniel Reichek, Madhavi Kadiyala, Kathleen Gliganic, Alistair Young

**Affiliations:** 1 St. Francis Hospital, Research and Education Foundation, Roslyn, NY USA; 2 Medicine, Stony Brook University, Stony Brook, NY USA; 3 Anatomy, University of Auckland, Auckland, New Zealand

## Background

All indices of systolic myocardial performance, including CMR strain, reflect the interplay between contractile state and loading conditions. However, CMR strains have been used largely in descriptive fashion, without consideration of physiologic determinants. We have previously demonstrated the role of increased afterload as a determinant of CMR strain using feature tracking (FT) in dilated cardiomyopathy as compared to normals, as well as the utility of an afterload index based on 3D volumetric geometric determinants (PV/M) compared to wall stress (WS) calculation. We have also shown the potential value of ratios of strain to afterload (S/A) as simple, practical contractility indices and that low dose dobutamine (Dob) can increase contractility, increasing both strain and strain rate without detectable change in blood pressure (BP) or heart rate (HR). However the relative sensitivity of CMR strain, S/A indices and strain rate to changes in contractile state and the performance of these indices with various CMR strain methods are unknown.

## Methods

In 6 carefully screened normal volunteers (5 male, mean age 47.1+/-6.8 yrs) we determined global circumferential and longitudinal strain (CSt,LSt) and strain rate at rest and during infusion of low dose DOB (1.9-2.5 mcg/kg/min), titrating to avoid changes in HR and BP in each subject. Volumetric CMR SSFP cine imaging, pre and post contrast T1 mapping for extracellular volume (ECV) determination, and DENSE strain imaging in short and long axis planes were obtained. FT and DENSE CSt, LSt and mean systolic strain rates, circumferential and meridional wall stress and PV/M index, were determined using cuff systolic BP, CMR LV mass and end systolic volume at rest and during DOB and results evaluated with t-tests and linear regression.

## Results

(Table 1) DENSE global longitudinal strain appears to be the strain index tested most reflective of increased contractile state, while feature tracking longitudinal strain/stress and DENSE circumferential and longitudinal strain/stress ratios are the most sensitive Strain/afterload indices. However, mean strain rate indices appear superior to S/A ratios in depiction of increased contractility. Finally, low dose dobutamine in normals elicits strong inverse correlations between PV/M and ECV and strain/afterload ratios and ECV.

**Table 1:** Table 1

Afterload			
CWS	202.4+/-39.7	172.1+/-60.2	ns
MWS	80.3+/-19.7	60.3 +/-27.5	ns
PV/M	87.6+/-22.2	76.2+/-16.6	ns
Strain			
FT CSt	-14.9+/-1.9	-15.8+/-2.4	ns
FTLSt	-13.95+/-1.5	-15.1+/-1.1	ns
DENSE CSt	-15.0+/-1.8	-16.4+/-2.9	ns
DENSE LSt	-7.2+/-2.4	-10.3+/-1.2	0.02
Contractility			
S/A Ratios			
FT CSt/CWS	-.077 +/-0.02	-.010 +/-0.03	ns
FT CSt/PV/M	-.18 +/-0.06	-.22 +/- 0.06	ns
FT LSt/MWS	-0.186+/-0.1	-0.288+/-0.1	0.04
FT LS/PV/M	-0.17 +/-1.5	-0.21+/- 1.1	ns
DENSE CSt/CWS	-.073+/-0.01	-.094+/-0.02	<0.001
DENSECST/PV/M	-0.17+/-0.05	-0.20+/-0.06	ns
DENSE LSt/MWS	-0.097+/- 0.05	-0.183+/-.070	ns
DENSE LSt/PV/M	-0.88+/- 0.04	-0.136+/-0.04	ns
Strain Rates			
FT CStR	-0.043+/-0.005	-0.053+/-0.009	0.023
FT LStR	-0.039 +/- 0.005	-0.051 +/-0.004	0.02
DENSE CStR	-0.04+/-0.006	-0.05+/-0.006	0.004
DENSE LStR	-.020+/-.0065	-.035+/-.0052	0.01
Correlations with ECV	r=	p=	
DOB PV/M vs. ECV	-0.82	0.027	
FT DOB LS/PV/M vs.ECV	-0.82	0.028	
DENSE LS/MWS vsECV	-0.87	0.013	

## Conclusions

Systolic mean strain rates with feature tracking or DENSE strain imaging are sensitive indices of contractile state. There is an inverse correlation between beta adrenergically generated increases in the global afterload index PV/M and myocardial extracellular volume as well as an inverse correlation between ECV and dobutamine induced increases in contractile indices based on the ratio of strain to afterload. This apparent effect of ECV differences within the normal range may be an important determinant of variability of normal contractile reserve.

